# MRPS30-DT Knockdown Inhibits Breast Cancer Progression by Targeting Jab1/Cops5

**DOI:** 10.3389/fonc.2019.01170

**Published:** 2019-11-07

**Authors:** Balu Wu, Yunbao Pan, Guohong Liu, Tian Yang, Yanxia Jin, Fuling Zhou, Yongchang Wei

**Affiliations:** ^1^Department of Hematology, Zhongnan Hospital of Wuhan University, Wuhan University, Wuhan, China; ^2^Department of Laboratory Medicine, Zhongnan Hospital of Wuhan University, Wuhan University, Wuhan, China; ^3^Department of Radiology, Zhongnan Hospital of Wuhan University, Wuhan University, Wuhan, China; ^4^Department of Clinical Oncology, Zhongnan Hospital of Wuhan University, Wuhan University, Wuhan, China; ^5^Hubei Key Laboratory of Tumour Biological Behaviors, Department of Radiation and Medical Oncology, Zhongnan Hospital of Wuhan University, Wuhan, China

**Keywords:** breast cancer, lncRNA, MRPS30-DT, Jab1, biomarker

## Abstract

Longnoncoding RNAs (lncRNAs) are significantly correlated with cancer pathogenesis, development, and metastasis. Microarray analysis showed that lncRNA MRPS30-DT is overexpressed in breast carcinoma; however, the function of MRPS30-DT in breast cancer tumorigenesis remains unclear. *In situ* hybridization and immunohistochemical analysis were used to evaluate the expression levels of MRPS30-DT and Jab1 in clinical samples of breast carcinoma and their relation to survival outcome. qRT-PCR was used to measure MRPS30-DT and Jab1 mRNA expressions. Protein levels were detected using Western blot. Cell proliferation and invasion ability were evaluated via 3-(4,5-dimethylthiazol-2-yl)-2,5-diphenyltetrazolium bromide (MTT), colony formation, and transwell assays. MRPS30-DT was knocked down in breast cancer cells to investigate its potential functional roles in cell growth and metastasis *in vitro* and *in vivo*. We found that MRPS30-DT was upregulated in breast cancer specimens and was accompanied by high Jab1 expression compared with that of paired para-carcinoma tissues. Knocking down MRPS30-DT significantly inhibited cancer cell proliferation and invasion and induced apoptosis in breast cancer cells. Similarly, knocking down MRPS30-DT in MDA-MB-231 cells significantly suppressed tumor growth. Furthermore, knocking down MRPS30-DT markedly reduced Jab1 expression in breast cancer cells and murine carcinoma. Statistical analyses suggested that high MRPS30-DT or Jab1 levels in breast cancer patients were positively correlated with poor prognoses. These data indicate the possible mechanisms of MRPS30-DT and Jab1 in breast cancer; thus, MRPS30-DT and Jab1 may be novel prognostic biomarkers and potential therapeutic targets for breast cancer treatment.

## Introduction

Breast cancer is one of the most common malignant tumors in women and is the leading cause of cancer-related death in women (1, 2). Epidemiological investigations have shown that genetic susceptibility and environmental factors and reproductive factors are closely related to breast cancer (3). The main treatments for breast cancer include surgery, chemotherapy, radiotherapy, targeted therapy, endocrine therapy, and comprehensive therapy (4). Despite advances in adjuvant therapy in recent years, the 5-year survival rate for patients with advanced breast cancer is only around 22% (5). Patients who die of metastatic breast cancer account for 90% of these deaths (5). Precision therapy suggests that different breast cancer subtypes may have different molecular phenotypes and thus require different treatment strategies (4). For example, HER2-positive patients have more prolonged survival after targeted anti-HER2 therapy (6). These features confer different predictive and reactive therapies for breast cancer patients. Hence, novel therapeutic targets for breast carcinoma are urgently needed.

Jab1 was initially identified as a c-Jun coactivator (also called COPS5or CSN5) and plays an essential role in DNA response, DNA repair, the cell cycle, apoptosis, proliferation, and signal transduction (7, 8). Jab1 is reported to be highly expressed in various tumors and is significantly negatively correlated with tumor survival (9, 10). Conversely, knocking down Jab1 can significantly reduce tumor cell proliferation and invasion ability (11). Long noncoding RNA (lncRNA) TBILA is reported to promote non-small-cell lung cancer by activating the S100A7/Jab1 signaling pathway (10). Our previous research demonstrated that Jab1 contributes to breast cancer progression (8, 12). However, few studies have reported on whether lncRNA can regulate Jab1 expression in breast cancer.

Noncoding RNAs do not encode proteins (13–15). LncRNAs are noncoding RNAs that are more than 200 nucleotides long (13). The major functions of lncRNA include regulating chromatin modification, gene transcription, protein translation, and ceRNA function (13). LncRNA can adjust gene expressions at the transcriptional, posttranscriptional, and epigenetic levels; thus, lncRNA is involved in cancer occurrence and progression (16). Increasing evidence has shown that lncRNA plays an essential regulatory role in various tumors (16) and is abundant in mammals. Although lncRNA cannot be translated into proteins, it can regulate gene expression levels through various pathways (17). Previous research has shown that lncRNAs play a role in regulating proliferation, invasion, metastasis, and apoptosis. Thus, the underlying mechanisms that lncRNAs use to regulate breast cancer should be investigated.

Data were collected using a high-throughput microarray to analyze the lncRNA and mRNA expression profiles in breast cancer and para-carcinoma tissues. MRPS30-DT and Jab1 expression profiles in breast tumor tissues were markedly higher than those in the adjacent mucosa, and higher expression levels of MRPS30-DT or Jab1 correlated with worse prognoses in breast cancer patients. Functional studies showed that MRPS30-DT significantly promoted tumor cell proliferation, induced tumor cell invasion and metastasis, and inhibited tumor cell apoptosis. Knocking down of MRPS30-DT significantly reduced Jab1 expression in breast cancer cell lines. Overall, these results indicated that MRPS30-DT plays an oncogenic role in breast cancer and may regulate breast cancer development by targeting Jab1. Our study characterized a novel lncRNA-mediated mechanism of regulating Jab1 and may provide a novel diagnostic marker and therapeutic target for treating breast cancer patients.

## Materials and Methods

### Microarray Analysis

Six pathological tissues (three pairs) obtained from breast cancer patients were included in the human microarray analysis using the Agilent Array platform (Agilent Technology) per the manufacturer's protocol. Differentially expressed lncRNAs were identified in breast cancer and para-carcinoma tissues via fold-change filtering, and a heatmap was used to plot the expression profiles. All three enrolled patients had pathologically confirmed invasive ductal carcinoma.

### Chemicals and Reagents

RPMI-1640 cell basal culture medium was purchased from Invitrogen (Carlsbad, CA, USA), and fetal bovine serum (FBS) was purchased from Gibco (Grand Island, NY, USA). The total RNA extraction kit and Lipofectamine 2000 transfection reagent were also purshased from Invitrogen (Carlsbad, CA, USA). Si-MRPS30-DT and the negative control (si-NC) were purchased from GenePharma (Shanghai, China). The anti-Jab1 antibody was purchased from Santa Cruz Biotechnology (Santa Cruz, CA, USA), and anti-GAPDH was purchased from Proteintech (Wuhan, China).

### Cell Lines and Cell Cultures

The human breast cancer cell lines (MDA-MB-231 and MCF-7) were provided by the Type Culture Collection of the Chinese Academy of Sciences (Shanghai, China). The cells were sustained in RPMI-1640 medium containing 10% FBS and 1% penicillin-streptomycin sulfate (Invitrogen). All cells were cultivated at 37°C in a 5% CO_2_ atmosphere.

### Tissue Microarray and Immunohistochemical Analyses

The tissue microarray was obtained from Outdo Biotech Co., Ltd. (Shanghai, China). Immunohistochemical studies of Jab1 and *in situ* hybridization analyses of MRPS30-DT were performed on the breast cancer samples via tissue microarray. The paraffin-embedded tissues were sliced at 4-μm thick. After dewaxing and rehydration, the tissue sections were incubated with 3% H_2_O_2_ for 30 min to block the endogenous peroxidase activity. The antigen was recovered through repeated cooling and heating, and nonspecific binding was blocked with 5% bovine serum albumin. The sections were incubated with primary antibodies overnight at 4°C. Anti-Ki67 (ab833) was purchased from Abcam (Cambridge, MA, USA). Anti-Ki67 was diluted at 1:200; anti-Jab1 was diluted at 1:50. The sections were washed three times with phosphate-buffered saline (PBS) for 5 min, then treated with biotinylated secondary antibody (Abcam) for 1 h and with streptavidin-horseradish peroxidase (HRP) for 20 min. Ki67- and Jab1-positive cells were stained using diaminobenzidine (DAB) substrate and observed under a microscope (Olympus BX51, Olympus Optical, Tokyo, Japan).

A digoxigenin (DIG)-labeled MRPS30-DT probe (Exiqon) was used to perform ISH staining on TMA. Histologic sections were hybridized with a dual probe-labeled RNA probe for 2 h, then detected with an anti-DIG antibody. Cancer cells were MRPS30-DT-positive when the cytoplasm or nucleus was stained.

### Cell Transfection and Transduction

The siRNA transfected using Lipofectamine 2000 (Thermo Fisher Scientific, Rockford, IL, USA) per the manufacturer's protocol. siRNA oligomers were synthesized by GenePharma (Shanghai, China). The MRPS30-DT_siRNA (#1) sequences were 5′-CUUCUCUGUAGUGUAUGCUTT-3′ and siRNA (#2) 5′-GGGUCUAUGGGUGUAUUTT-3′, and the control si-NC sequence was 5′-UUCUCCGAACGUGUCACGUTT-3′. MCF-7 or MDA-MB-231 cells were seeded into six-well plates (150,000 cells/well) overnight, then transfected with siRNA (#1), siRNA (#2), or si-NC. Cells were used for further tests 24–48 h after transfection. Lentivirus transfection techniques were used to establish stable cell lines. Briefly, a short hairpin RNA (shRNA) targeting MRPS30-DT was constructed into a lentivirus vector (shMRPS30-DT-#1, shMRPS30-DT-#2). A lentivirus vector carrying a nonspecific sequence was used as a negative control. The viruses were packaged in 293T cells, and the virus particles were harvested and filtered 72 h after transfection. Target cells were cultured in serum-containing medium with virus particles with 1.2 μg/ml polybrene. Stably transfected cells were selected by culturing in medium containing 0.8 μg/ml puromycin (Sigma-Aldrich, St. Louis, MO, USA).

### RNA Extraction and Real-Time PCR

Total RNA from MCF-7 and MDA-MB-231 cells was isolated with Trizol reagent (Invitrogen and Thermo Fisher Scientific) per the manufacturer's protocol. The purity and concentration of the total RNA were measured using a NanoDrop ND-2000 spectrometer (NanoDrop Technologies, Wilmington, DE, USA). Total RNA (500 ng) was reverse transcribed using a Reverse Transcription Kit (Takara, Dalian, China). qRT-PCR was performed using an Applied Biosystems 7500 system (Applied Biosystems, Foster City, CA, USA). As specified by the PrimeScriptTM RT Master Mix (Perfect Real-Time) kit, cDNA was compounded and used for real-time fluorescence qPCR. The qRT-PCR reaction system (10 μl) comprised 5 μl SYBR qPCR Mix, 0.5 μl (10 μmol/L) upstream primer, 0.5 μl (10 μmol/L) downstream primer, and 2 μl cDNA product; RNase-free water was added to 10 μl. The thermocycling conditions were denaturation at 95°C for 10 min, 95°C for 10 s, annealing at 60°C for 40 s, and extension at 72°C for 30 s for 40 cycles. The primer sequences were as follows: MRPS30-DT, forward 5′-ATT CCA GCC ACT CCA TTT CTA-3′ and reverse 5′- GAC CCT ATA CGG CAA CCT CCT-3′; Jab1, forward 5′-CGG TAT GGC CCA GAA AAC CT-3′ and reverse 5′- CTT CCA AGT TGC CTC CCG AT-3′; and GAPDH, forward 5′-GAA GGT GAA GGT CGG AG TC-3′ and reverse 5′-GAA GAT GGT GAT GGG ATT TC-3′. GAPDH served as an endogenous control to normalize MRPS30-DT and Jab1 expression. The relative quantities of MRPS30-DT and Jab1 were counted using the 2−ΔΔCq method.

### Western Blotting

For the Western blot, the appropriate volume of cell lysis buffer was added to the treated cells or samples for lysis on ice and supernatant was collected after centrifugation. The protein concentration was measured using a bicinchoninic acid (BCA) protein assay kit (Thermos, Waltham, MA, USA). Fifteen micrograms of proteins were separated using 12% SDS-PAGE, then the gels were subsequently transferred onto 0.22-μm PVDF membranes (Millipore Corp., MA, USA), and the membranes were blocked with 5% skim milk for 1 h at room temperature. The blot was then probed with mouse monoclonal antibodies against anti-Jab1 (1:750, Santa Cruz). As primary antibodies, anti-GAPDH was used as the internal positive control for the immunoblots and was incubated at 4°C overnight. Subsequently, the membranes were washed three times for 10 min and incubated with HRP-conjugated goat anti-mouse IgG (1:5,000; Sigma) as secondary antibodies for 1 h at room temperature. Specific protein bands were detected using a Western Chemiluminescent Imaging System (Tanon5200, Wuhan, China).

### Cell Proliferation Assay

The 3-(4,5-dimethylthiazol-2-yl)-2,5-diphenyltetrazolium bromide (MTT) assay was used to assess the cell's ability to proliferate. Briefly, 48 h after transfection, the cells were seeded at 1 × 10^3^ cells/well in 96-well plates. Twenty microliters of MTT solution (0.5 mg/ml) was added to each well at 1, 2, 3, 4, 5, and 6 days after transfection. The cells were incubated at 37°C for 4 h, then 150 μl dimethyl sulfoxide (DMSO) was added to each well. The absorbance was read at 570 nm using a multifunctional enzyme-linked immunosorbent assay microplate reader (SpectraMax M2, CA, USA). The experiments were repeated at least three times.

### Colony-Formation Assay

For the colony-formation assay, the transfected cells were seeded in six-well plates at 200 cells/well. The culture medium was changed twice weekly. The breast cancer cells were cultured for 10–14 days at 37°C, then fixed and stained with 0.1% crystal violet. The colonies (one colony consisted of 50 or more cells) were scored by counting with an inverted microscope. Cell viability was calculated as the number of colonies in the treatment group/the number of colonies in the control group × 100%.

### Migration and Invasion Assay

A wound-healing assay was used to assess the migration capacity of the breast cancer cells. Briefly, the transfected cells were seeded and cultured in six-well plates (5 × 10^5^ cells per well), and when the cell density was near 90%, the confluent cell monolayer was scratched in a straight line using a 200-μl plastic pipette tips and washed with sterile PBS. Cells were further cultured with medium containing 1% FBS for 48 h. Scratch closures were photographed at 0, 12, 24, and 36 h under a light microscope (Olympus IX50, Tokyo, Japan). Image-Pro Plus software (Version 5.1, Media Cybernetics, Inc., Maryland, USA) was used to calculate the cellular migration ability in each group.

Invasion assays were performed in 24-well plates precoated with Matrigel (BD, Franklin Lakes, NJ, USA). After transfection, MCF-7 or MDA-MB-231 cells (at 5 × 10^3^ cells/well in 100 μl of serum-free media) were seeded in the upper chamber, and 600 μl of medium containing 10% FBS was placed in the lower chamber. The cells were incubated for 24 h at 37°C. Cells in the upper chambers were removed with a swab, and the invaded cells were fixed with methanol for 15 min, then stained with 0.1% crystal violet and scored by counting with an inverted-contrast microscope using at least five random fields of view. The experiments were repeated at least three times.

### Flow Cytometry Analysis of Apoptosis

Cell apoptosis was evaluated using the Annexin V-FITC/PI Apoptosis Detection Kit (Kaiji, Nanjing, China). Treated cells were harvested by low-speed centrifugation (900 r/min) and washed twice with ice-cold PBS. Next, 500 μl of binding buffer was added to the suspended cells, and cells were stained with 5 μl propidium iodide (PI) and 5 μl Annexin V-FITC at room temperature for 15 min in the dark. Flow cytometry (Becton Dickinson, CA, USA) was used to test apoptosis. Finally, the results were interpreted using FlowJo software, version 10.0 (Flow Jo, Ashland, OR, USA).

### Animal Experiments

Five-week-old female BALB/C nude mice (*n* = 12) were obtained from Beijing HFK Bioscience (Beijing, China) and raised in a specific pathogen-free, climate-controlled facility (Wuhan, China). The mice were randomly distributed into three groups (four mice per group). MDA-MB-231 cells treated with sh-MRPS30-DT or sh-NC were subcutaneously injected into the right or left flank, respectively (200 μl, 5 × 10^6^ cells per mouse). The mouse weights and tumor volumes were measured twice weekly. Tumor volume was calculated per the formula: Tumor volume (mm^3^) = Tumor length (mm) × Tumor width (mm)^2^/2. Five weeks later, the mice were humanely killed, and the tumors were extracted and weighed. The appropriate amount of tumor tissue was taken for paraffin embedding and immunohistochemical analysis. The experiments were implemented under the protocol approved by the institutional and national guidelines for the care and use of laboratory animals.

### Statistical Analysis

Statistical analysis between two groups was performed using Student's *t*-test. One-way ANOVA was used for between-group comparisons. Kaplan-Meier analysis was used to evaluate the association between MRPS30-DT and Jab1 expression and survival. Differences between groups were considered statistically significant at *P* < 0.05. All computations were performed using GraphPad 7.0 and SPSS22.0 software.

## Results

### Microarray Expression Profiles

**Figure 1:**
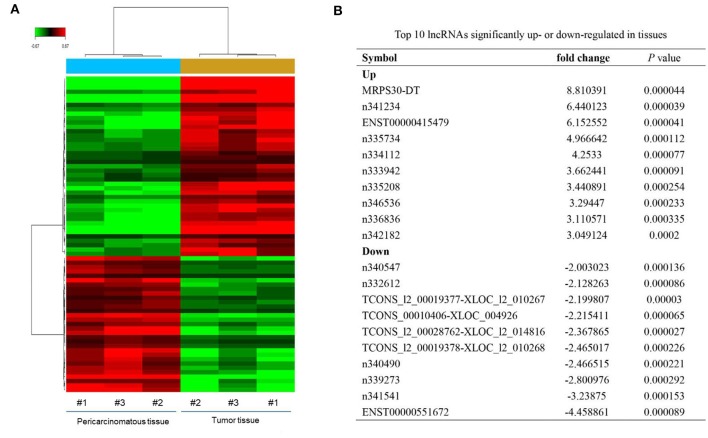
LncRNAs expression profiling in three paired breast cancer and adjacent normal tissues. **(A)** The heatmap reveals clusters of differential expressed lncRNAs. The green color indicates downregulated lncRNAs, and the red color indicates upregulated genes. **(B)** Top 10 lncRNAs significantly upregulated or downregulated in tumor tissue.

Microarray analysis revealed 52 significantly dysregulated lncRNAs (fold-change ≥ 1.5, *P* < 0.001) in the profiles. Thirty-three upregulated and 19 downregulated lncRNAs showed significantly different expressions between the breast cancer tissues and adjacent normal tissues (Figure 1A). Figure 1B summarizes the top 10 upregulated and downregulated genes. MRPS30-DT was most significantly upregulated in the tumor tissue; thus, we chose it for further studies.

### MRPS30-DT and Jab1 Expression in Breast Cancer Tissue

**Figure 2:**
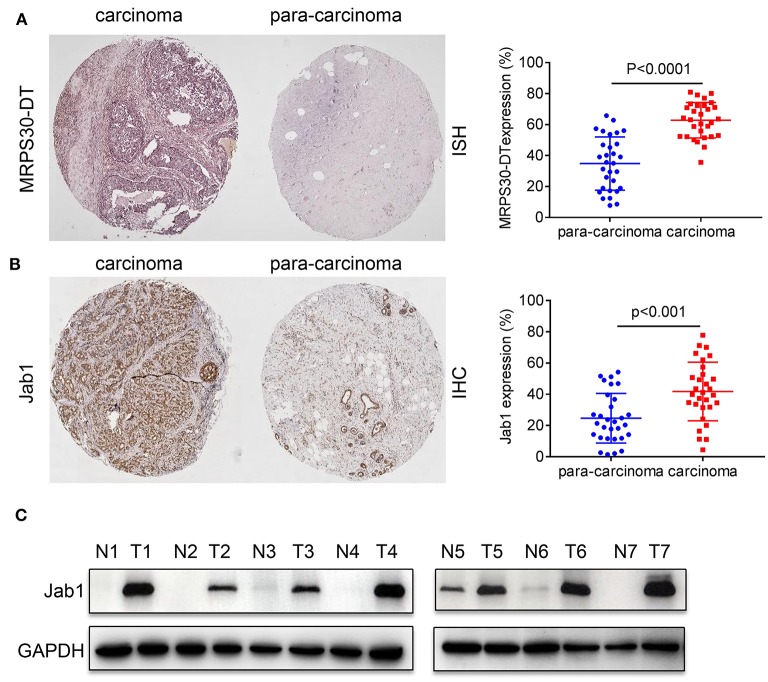
Expression correlation analysis of lncRNA MRPS30-DT and Jab1 in breast cancer tissues and matched adjacent noncancerous tissues. **(A)** *In situ* hybridization (ISH) demonstrating MRPS30-DT in breast cancer tissues was higher than that in noncancerous tissues (*n* = 30, *p* < 0.0001). **(B)** Jab1 immunoreactivity in breast cancer tissues was higher than that in noncancerous tissues. The percentage of Jab1 expression in breast cancer or noncancerous tissues are shown in the figure (*n* = 30, *p* < 0.001). The patient population represented were from Outdo Biotech Co., Ltd. (Shanghai, China). **(C)** Jab1 proteins have higher expression in breast cancer tissues. The expression levels of Jab1 proteins in human breast cancer and paired adjacent noncancerous tissues from seven random clinicallydiagnosed breast cancer patients were measured by Western blot analysis. GAPDH was used as a loading control. N, paired adjacent normal tissues; T, tumor tissues. Patient information is placed in Supplementary Materials.

We used *in situ* hybridization and immunohistochemical analysis to evaluate the differential expressions of MRPS30-DT and Jab1 in the breast cancer and matched adjacent tissues. The *in situ* hybridization results showed that MRPS30-DT was positively expressed in 63% of breast cancer samples. This ratio was significantly higher than that of the paired adjacent noncancerous breast tissue specimens (*P* < 0.0001; Figure 2A). Likewise, immunohistochemical analysis showed higher Jab1 expression levels in breast cancer tissues (42%) than in those of the adjacent noncancerous tissues (24%; *P* < 0.001; Figure 2B). We used seven pairs of tumor tissues and matched adjacent tissues, and the results showed significantly higher Jab1 expression in tumor tissues than in the adjacent tissues (Figure 2C).

### MRPS30-DT Knockdown Inhibited Cell Proliferation and Induced Cell Apoptosis

**Figure 3:**
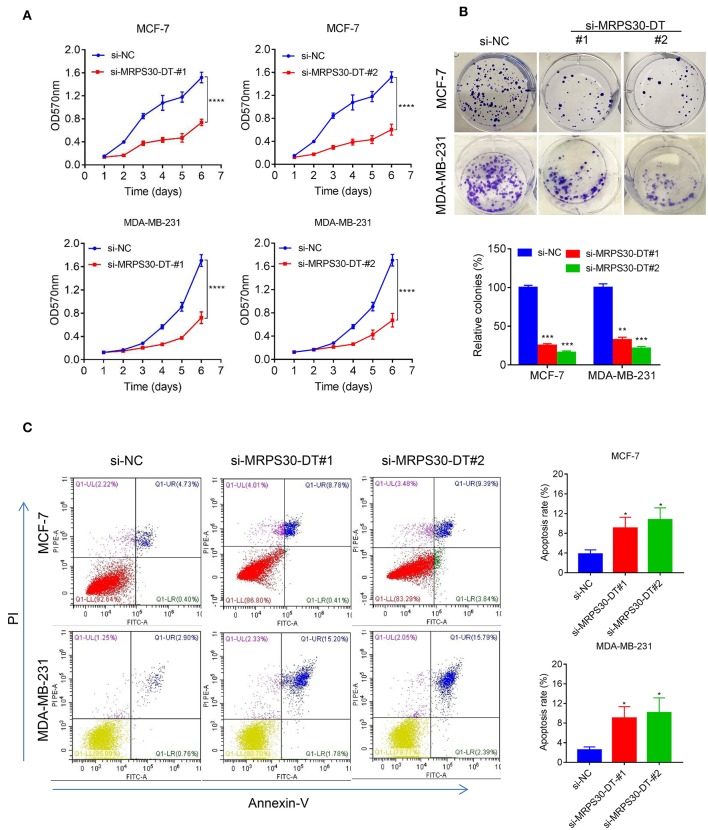
MRPS30-DT knockdown inhibits cell proliferation and promotes apoptosis of breast cancer (BC) cells. **(A)** Breast cancer cell lines (MCF-7 and MDA-MB-231) were transfected with negative control (NC) or si-MRPS30-DT-#1 or si-MRPS30-DT-#2 for 48 h, and cell growth was determined via MTT assay. The quantitative values of cell viability were shown by the mean OD value ± SD. **(B)** Downregulation of MRPS30-DT suppressed BC cells proliferation via colony formation assay. The quantitative values of cell clonality were shown by the mean percent of means ± SD. **(C)** Apoptotic cell death was detected by flow cytometric analysis with Annexin V-FITC and PI staining in MCF-7 and MDA-MB-231 cells transfected with MRPS30-DT NC or siRNA for 48 h. The experiment was repeated three times (*P < 0.05, **P < 0.01, ***P < 0.001, ****P < 0.0001).

MCF-7 and MDA-MB-231 cells were transfected with si-NC RNAs and MRPS30-DT siRNAs for 48 h, respectively. MTT assays showed that MRPS30-DT knockdown markedly inhibited the proliferative ability of breast cancer cells compared with that of the control group (Figure 3A), and the colony-formation experiment results were consistent with the MTT assay results (Figure 3B). Furthermore, flow cytometry revealed that knocking down MRPS30-DT promoted cell apoptosis (Figure 3C). Thus, MRPS30-DT knockdown inhibited proliferation and accelerated apoptosis in breast cancer cells.

### Knocking Down MRPS30-DT Suppresses Cell Migration and Invasion

**Figure 4:**
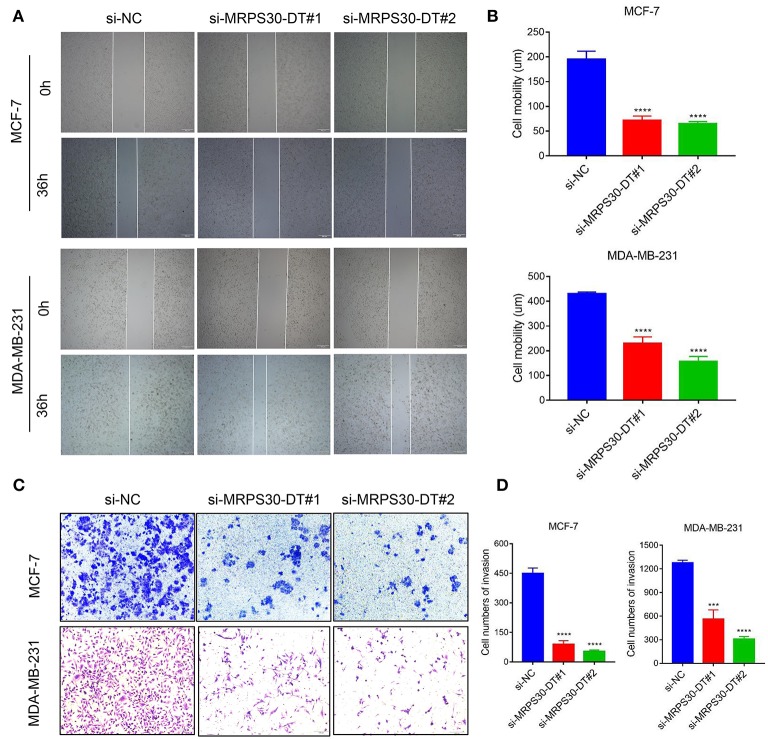
Inhibition of MRPS30-DT decreased cell migration and invasion. **(A,B)** Cell migration was measured by scratch-wound assay. Representative images are shown (20×). The results of three independent experiments are summarized; **(C,D)** Cell invasion was determined by transwell assay. Representative images are shown (100×). The results of three independent experiments are shown (***P < 0.001, ****P < 0.0001).

We used wound healing and transwell assays to investigate how MRPS30-DT influenced breast cancer cell migration and invasion. The wound-healing assay showed that knocking down MRPS30-DT significantly decreased the cell migration distance in MCF-7 and MDA-MB-231 cells (Figures 4A,B). Transwell assays indicated that MRPS30-DT positively regulated cell invasion.Figures 4C,D show that inhibiting MRPS30-DT significantly suppressed cell invasion in MCF-7 and MDA-MB-231 cells. These data illustrate that MRPS30-DT facilitated breast cancer cell migration and invasion.

### MRPS30-DT Positively Regulated Jab1/Cops5 in Breast Cancer

**Figure 5:**
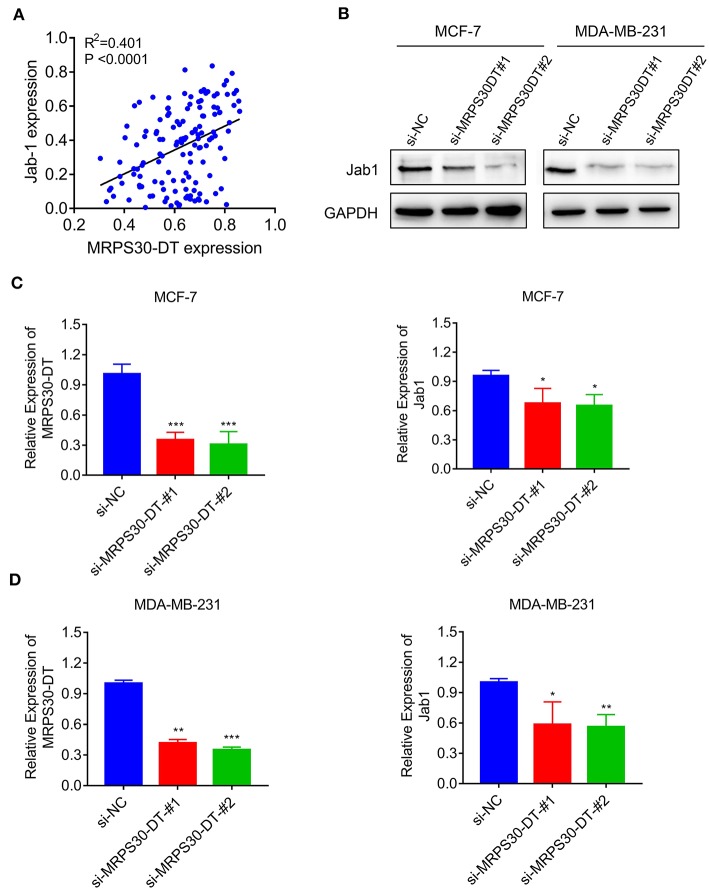
MRPS30-DT targets Jab1. **(A)** The expression of MRPS30-DT was correlated with Jab1 expression in breast cancer from the TMA data (*n* = 140), the patient population represented were from Outdo Biotech Co., Ltd. (Shanghai, China). The *R*^2^ and *P-*values were from Pearson Correlation. **(B)** Western blot results of Jab1 protein level by transfecting with si-NC or si-MRPS30-DT. GAPDH was used as an internal control. **(C,D)** qRT-PCR analysis of MRPS30-DT expression and Jab1 expression after si-NC or si-MRPS30-DT were transfected for 24 or 48 h (*P < 0.05; **P < 0.01; ***P < 0.001).

The TMA data revealed that MRPS30-DT expression was correlated with Jab1 in breast cancer patients (*R*^2^ = 0.401, *P* < 0.0001; Figure 5A). To further understand the effects of MRPS30-DT on breast cancer cells, siRNA was used to downregulate the MRPS30-DT expression in the MCF-7 and MDA-MB-231 cells. Transfection of MRPS30-DT siRNA decreased the Jab1 expression in MCF-7 and MDA-MB-231 cells (Figures 5B–D). These data indicated that Jab1/COPS5 were strongly positively correlated with MRPS30-DT in breast carcinomas. Thus, Jab1 may be a target of MRPS30-DT.

### Knocking Down MRPS30-DT Inhibited Tumorigenesis *in vivo*

**Figure 6:**
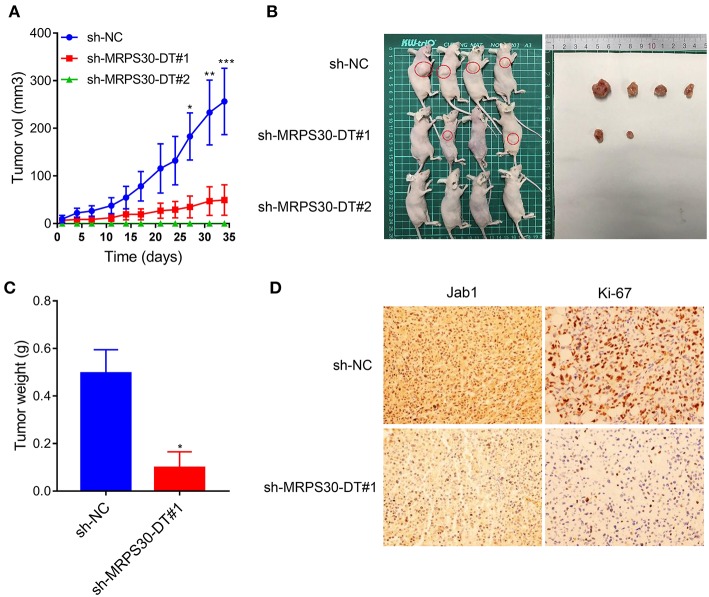
Knockdown of MRPS30-DT significantly suppressed tumorigenesis *in vivo*. **(A,B)** After stable transfection of sh-NC or sh-MRPS30-DT in MDA-MB-231 cells, cells are harvested and subcutaneously injected into the right or left flank of female nude mice (*n* = 4), 5 weeks later, nude mice were executed humanely. Tumors are taken for photographing. **(C)** Tumor masses are weighed after being dissected. The indicated tumor volumes and tumor weights represent the mean ± SD. **(D)** Representative photomicrograph of Jab1 and Ki67 immunostaining in MDA-MB-231 xenograft tumor (*P < 0.05; **P < 0.01; ***P < 0.001).

To validate the findings *in vivo*, MDA-MB-231 cells were infected with lentivirus carrying shRNA-MRPS30-DT or negative control shRNA, then the cells were subcutaneously injected into BALB/C nude mice to establish xenograft models. After 5 weeks, the tumor volume in the shRNA-MRPS-DT group was dramatically reduced, and the tumor weights were less than those of the control group (Figures 6A–C). Immunohistochemical analysis of Jab1 and Ki67 expression was detected in the xenograft tumors, and the positive rates of Jab1 and Ki67 were remarkably lower compared with the those of control group (Figure 6D). Thus, knocking down lncRNA MRPS30-DT inhibited MDA-MB231 cell growth *in vivo*.

### Correlation of MRPS30-DT/Jab1 Expression With Clinical Outcomes

Breast cancer patients with high MRPS30-DT expression had a significantly shorter median survival (113 months) than did those with low MRPS30-DT expression (125 months). Likewise, the mean survival time of patients with highly positive Jab1/COPS5 tumors (116 months) was shorter than that of patients with weakly positive Jab1/COPS5 tumors (122 months).

**Figure 7:**
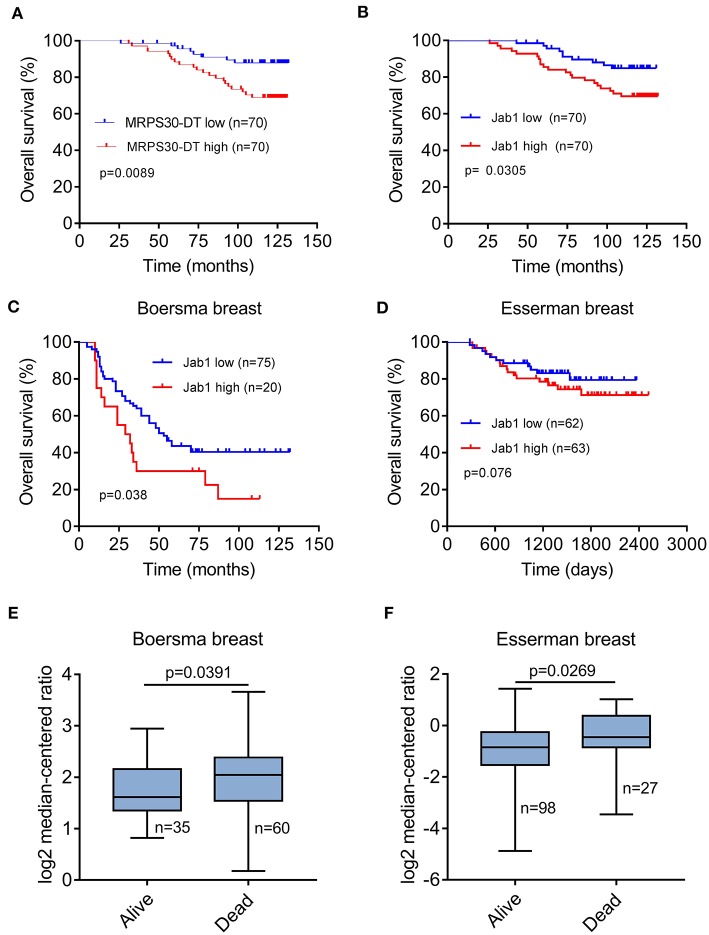
MRPS30-DT and Jab1 predict survival in breast cancer. **(A)** Kaplan-Meier analysis of the association between MRPS30-DT expression and overall survival. **(B)** Kaplan-Meier analysis of the association between Jab1 expression and overall survival. The patient population represented in **(A,B)** were from Outdo Biotech Co., Ltd. (Shanghai, China). **(C–F)** Jab1 expression in breast cancer (BC) in different clinical research centers using the Oncomine gene expression tool (https://www.oncomine.com). The clinical data were downloaded from Oncomine Data Portal; Kaplan-Meier analyses of the association between Jab1 expression and overall survival in Boersma breast **(C)** and the association between Jab1 expression and survival in Esserman breast **(D)**. Jab1 gene expression in Boersma breast **(E)** and Esserman breast **(F)** between alive and dead.

Kaplan-Meier survival analysis showed that MRPS30-DT expression was significantly negatively correlated with overall survival (OS) of breast cancer patients (*P* = 0.009; Figure 7A). Likewise, increased Jab1 expression was associated with shorter OS (*P* = 0.037; Figure 7B). The Oncomine gene expression tool (https://www.oncomine.com) similarly showed that higher Jab1 expression was correlated with shorter OS of breast cancer patients (Figures 7C–F). These results indicated that MRPS30-DT and Jab1 were overexpressed in breast cancer and may be potential prognostic biomarkers in breast cancer.

## Discussion

Breast cancer is one of the most common malignancies in women worldwide and poses severe threats to public health (5, 18). Despite remarkable progress in diagnosing and treating breast cancer, the long-term outcomes and OS remain very poor (19, 20). One of the leading causes of death in breast cancer patients is tumor cell metastasis mainly because of the lack of early biomarkers (21–23). Therefore, molecular markers that can diagnose and monitor metastasis early are urgently needed.

Noncoding RNAs account for more than 90% of the total human gene transcripts and participate in nearly all epigenetic regulation, which is of great significance in the life activities of advanced eukaryotes (17, 24). Rapidly developing second-generation sequencing technology has yielded much evidence suggesting that lncRNA dysregulation is involved in cancer progression. Some studies have reported that various lncRNAs act as oncogenes or tumor suppressor genes in breast cancer. For example, Xing et al. reported that lncRNA YIYA promotes cell proliferation and growth by regulating breast cancer cell glycolysis (25). Gupta et al. demonstrated that the lncRNA HOTAIR promotes breast cancer progression and metastasis by recruiting PRC2 complex to specific targets genes genome-wide, leading to H3K27 trimethylation and epigenetic silencing of metastasis suppressor genes (26). Meng et al. used bioinformatics to show that four lncRNAs are highly expressed in breast cancer and were significantly positively correlated with OS and metastasis (27). Peng et al. discovered that lncRNA NEAT1 is upregulated in breast cancer and promotes cancer progression via the NEAT1/miR-124/STAT3 axis (28). Increasing evidences have testified that lncRNAs can regulate the expression of their adjacent genes *in cis* or modulate gene transcription *in trans* through epigenetic, transcriptional, and posttranscriptional mechanisms (29). By base pairing, lncRNAs can directly interact with DNAs or RNAs and form a strong duplex or a triplex (30). Highly structured lncRNAs can provide protein binding sites as well, forming ribonucleic-protein complexes with chromatin regulatory factors (30, 31).

MRPS30, also called programmed cell death protein 9 (PDCD9), encodes a mitochondrial ribosomal protein involved in apoptosis (32). MRPS30 is not expressed in normal breast luminal epithelial cells but is upregulated in infiltrating ductal carcinomas (33, 34). MRPS30 can affect ATP production to stimulate tumor growth (35). MRPS30-DT on chromosome 5p12, also called breast cancer-associated transcript 54 (BRCA54), is broadly expressed in the brain, thyroid and other tissues. Until now, no data have been found on the association between MRPS30-DT and breast cancer. The present study is the first report the role of MRPS30-DT in breast cancer.

Here, we found that MRPS30-DT expression levels were significantly higher in breast cancer tissues than in matched adjacent normal tissues and were positively correlated with a poor prognosis for breast cancer patients. Thus, MRPS30-DT may be highly expressed in breast cancer as an oncogene and may be a target for diagnosing or treating breast cancer. To further study this protein's effect on breast cancer cell proliferation, apoptosis, migration, and invasion ability, we downregulated MRPS30-DT in two selected breast cancer cell lines (MCF-7 and MDA-MB-231). Knocking down MRPS30-DT suppressed cell proliferation, migration, and invasion in these breast cancer cells while inducing apoptosis. In addition, we used lentivirus transfection technology to stably screen inhibitory strains for tumor formation in nude mice and found that inhibiting MRPS30-DT in MDA-MB-231 cells significantly inhibited tumor formation. These data further confirmed MRPS30-DT as an oncogene in breast cancer occurrence and development.

Jab1/COPS5 is highly expressed in various tumors, including nasopharyngeal carcinoma, non-small-cell lung cancer, liver cancer, colon cancer, and breast cancer (7, 36). Jab1/COPS5 expression is closely related to tumor progression and prognosis in many cancer patients and plays a vital role in cell proliferation, apoptosis, the cell cycle, DNA repair, and regulation of genomic stability (7, 8, 37). Jab1/COPS5 knockdown significantly inhibits proliferation and induces apoptosis in hepatocellular carcinoma cells (38). Our previous research explored the expression levels and mechanisms of action of Jab1 in various tumors. Jab1 regulates tumor progression by interacting with proteins or binding to miRNAs (11, 39). Our previous studies showed that Jab1/COPS5 was highly expressed in breast cancer and played an essential role in the breast cancer pathogenesis, and that Jab1 knockdown significantly inhibited breast cancer cell proliferation and metastasis (12). Here, the expression level of MRPS30-DT in breast cancer patients was positively correlated with Jab1.

Various mechanisms have been implicated in the lncRNA-mediated gene regulation, which can be attributed to their ability to interact with DNAs, RNAs, or proteins (40). For example, lncRNA may serve as signals to promote transcription, or as decoys to repress transcription, or as epigenetic regulators, or as scaffolds to interact with various protein partners to form ribonucleoprotein complexes (41–43). In our study, MRPS30-DT-mediated Jab1 expression took place at transcriptional and/or posttranscriptional levels; however, the detailed mechanism needs to be further studied.

In conclusion, this study demonstrated that MRPS30-DT was upregulated in breast cancer and was positively correlated with a poor prognosis in breast cancer patients. Knockdown of MRPS30-DT suppressed cell growth *in vitro* and *in vivo* and decreased cell migration and invasion abilities. Our study is the first evidence that MRPS30-DT plays an oncogenic role in breast cancer cell lines by targeting Jab1. Therefore, the carcinogenic effect of MRPS30-DT on breast cell tumorigenesis observed in this study could be partially attributed to its modulation of Jab1. This study aimed to explore the possible molecular mechanisms underlying the role of MRPS30-DT in the progression of breast carcinoma to provide new strategies to treat these tumors. Thus, MRPS30-DT may be a potential diagnostic and prognostic marker of breast cancer and a potential valuable therapeutic target in treating breast cancer.

## Data Availability Statement

The raw data supporting the conclusions of this manuscript will be made available by the authors, without undue reservation, to any qualified researcher.

## Ethics Statement

The studies involving human participants were reviewed and approved by Ethics Committee of Wuhan University. The patients/participants provided their written informed consent to participate in this study. The animal study was reviewed and approved by The Center for Animal Experiment of Wuhan University.

## Author Contributions

BW, YP, TY, and YJ performed the *in vitro* assays. BW, YP, and TY did the *in vivo* studies. GL and YP collected clinical samples. BW and YP analyzed the data and wrote manuscript. YP and YW designed this study. FZ and YW reviewed the manuscript. All authors read and approved the final manuscript.

### Conflict of Interest

The authors declare that the research was conducted in the absence of any commercial or financial relationships that could be construed as a potential conflict of interest.
