# Supplementary material for: MRPS30-DT Knockdown Inhibits Breast Cancer Progression by Targeting Jab1/Cops5
Source: Front Oncol. 2019 Nov 7;9:1170. doi: 10.3389/fonc.2019.01170 (PMC6854119; doi:10.3389/fonc.2019.01170)
Supplement: Supplementary file 1 [file Data_Sheet_1.PDF]

Tab1 Clinical information of human samples used in microarray.

Samples	Gender	Age	pathology type	Molecular type	T stage
1	Female	59	invasive ductal carcinoma	her2+/ER+	2
2	Female	42	invasive ductal carcinoma	Luminal	3
3	Female	60	invasive ductal carcinoma	Luminal	3

her2, human epidermal growth factor receptor-2; ER, estrogen receptor,

Tab2 Clinical information of human samples used for western blot.

Samples	Gender	Age	pathology type	Molecular type	T stage
1	Female	45	invasive ductal carcinoma	her2+	1
2	Female	53	invasive ductal carcinoma	Luminal	3
3	Female	32	invasive ductal carcinoma	Luminal	1
4	Female	75	invasive ductal carcinoma	Luminal	1
5	Female	57	invasive ductal carcinoma	Luminal	2
6	Female	40	invasive ductal carcinoma	her2+	2
7	Female	82	invasive ductal carcinoma	her2+	1

Figure S1

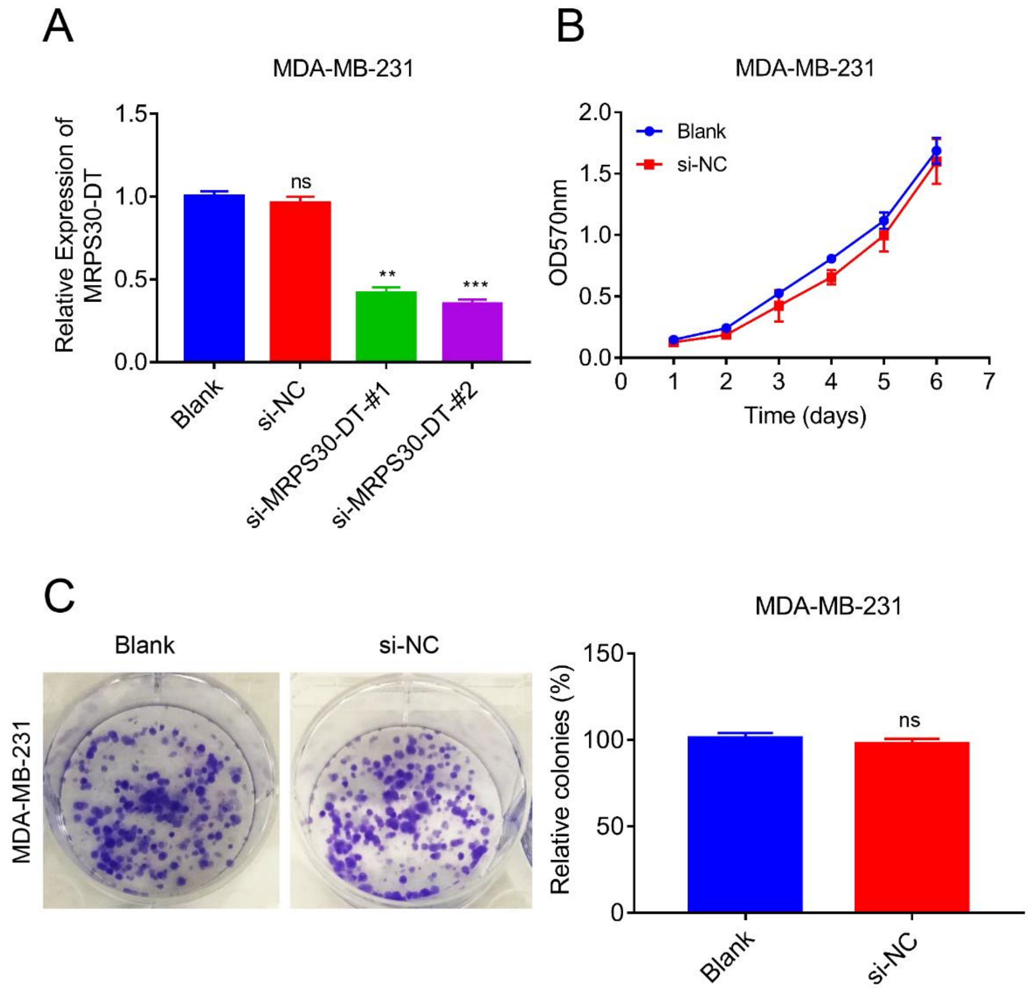

Figure S1

(A) The expression effects of silence of lncRNA-MRPS30-DT mRNA expression by siRNA in MDA-MB-231 cells. Cells were transfected with 50 μM negative control (NC) siRNAs and 50 μM lncRNA- MRPS30-DT siRNA for 24 h respectively, total RNA was isolated, qRT-PCR showed that expression levels of lncRNA-MRPS30-DT compared to negative control and blank. (n=3; $**P<0.01$, $***P<0.001$). (B) MTT assay showed that the transfected NC group had no effect on the proliferation of breast cancer cells. The cell viability assay was accomplished by MTT at six different durations. The quantitative values of cell viability are showed by the mean OD value of means ± SD (n=3). (C) Clone formation assay showed that NC transfection had no effect on proliferation of breast cancer cells.
